# Aortic root aortopathy in bicuspid aortic valve associated with high genetic risk

**DOI:** 10.1186/s12872-021-02215-y

**Published:** 2021-08-30

**Authors:** Mingjia Ma, Zongzhe Li, Mohamed Abdulkadir Mohamed, Ligang Liu, Xiang Wei

**Affiliations:** 1 Division of Cardiothoracic and Vascular Surgery, Tongji Hospital, Tongji Medical College, Huazhong University of Science and Technology, 1095# Jiefang Ave., Wuhan, 430030 People’s Republic of China; 2 Division of Cardiology, Departments of Internal Medicine and Genetic Diagnosis Center, Tongji Hospital, Tongji Medical College, Huazhong University of Science and Technology, Wuhan, People’s Republic of China; 3 Hubei Key Laboratory of Genetics and Molecular Mechanisms of Cardiologic Disorders, Wuhan, People’s Republic of China

**Keywords:** Bicuspid aortic valve, Next generation sequencing, Aortopathy, Rare variant

## Abstract

**Background:**

The bicuspid aortic valve (BAV) is prone to ascending aortic dilatation (AAD) involving both the tubular segment and the aortic root. The genetic factor was proposed as one of the most important mechanisms for AAD. We hypothesized that the rare genetic variants mainly contribute to the pathogenesis of aortic roots in affected individuals.

**Methods:**

The diameter of aortic root or ascending aorta ≥ 40 mm was counted as AAD. The targeted next-generation sequencing of 13 BAV-associated genes were performed on a continuous cohort of 96 unrelated BAV patients. The rare variants with allele frequency < 0.05% were selected and analyzed. Variants frequency was compared against the Exome aggregation consortium database. The pathogenicity of the genetic variants was evaluated according to the American College of Medical Genetics and Genomics guidelines.

**Results:**

A total of 27 rare nonsynonymous coding variants involving 9 genes were identified in 25 individuals. The burden analysis revealed that variants in *GATA5*, *GATA6*, and *NOTCH1* were significantly associated with BAV. Eighty percent of the pathogenic variants were detected in root group. The detection rate of rare variants was higher in root dilatation group (71.4%) compared with normal aorta (29.0%) and tubular dilatation groups (29.6%) (*P* = 0.018). The rare variant was identified as the independent risk factor of root dilatation [*P* = 0.014, hazard ratio = 23.9, 95% confidence interval (1.9–302.9)].

**Conclusions:**

Our results presented a broad genetic spectrum in BAV patients. The rare variants of BAV genes contribute the most to the root phenotype among BAV patients.

**Supplementary Information:**

The online version contains supplementary material available at 10.1186/s12872-021-02215-y.

## Background

Bicuspid aortic valve (BAV) is a common congenital valvular defect, affecting about 0.5–1% of the general population [1, 2]. Nearly half of the BAV affected patients would undergo medical or surgical management during their lifetime due to the BAV-related valvular and/or aortic complications (i.e. aortic stenosis, aortic insufficiency, aortic aneurysm, and aortic dissection et al.) [3]. Patients with BAV had a high risk of development of the ascending aorta dilatation (to a size above 4.0 cm) [4]. Of them, about 15% of patients have a dilated aortic root frequently with aortic insufficiency at a young age [5]. According to a large community cohort, the risk of aneurysm formation is significantly higher in BAV patients than the general population, and about a quarter of the patients with BAV may ultimately underwent aortic surgery after BAV diagnosis. The risk of acute aortic emergencies, most commonly aortic dissection, is eightfold in patients with BAV disease compared with the general population [6].

The underlying mechanisms of BAV-associated aortopathy are widely discussed. There are two potentially competing or more likely complementary perspectives of the cause of BAV aortopathy, i.e. the hemorheological reason and genetic factors. The former argues that the fused-leaflet of BAV alters the blood flow and increases the shear stress in the tubular ascending aorta [7]. From the genetic view, the genetic defect may increase the risk of BAV aortopathy [8]. However, which kind of influence would play a more important role is still controversial.

The aortic valve and ascending aorta share some common embryological origin [9]. It is reasonable to hypothesize that rare variants of BAV associated genes are likely to be risk factors predisposing to BAV aortopathy, especially in the aortic root. Human BAV presenting together with aortopathy has been proved to be associated with rare variants in *NOTCH1*, *TGFBR2*, *FBN1,* and *SMAD6 *et al. [5, 10]*.*

However, our knowledges of the inherited components affecting the clinical phenotype of BAV aortopathy are still limited. It could be argued that different types of BAV aortopathy may have different rare variants spectrum. By targeted next generation sequencing (NGS) of genes associated with BAV, we aimed to investigate distribution of rare variants in a large well-phenotyped BAV cohort.

## Methods

### Study population

**Fig. 1:**
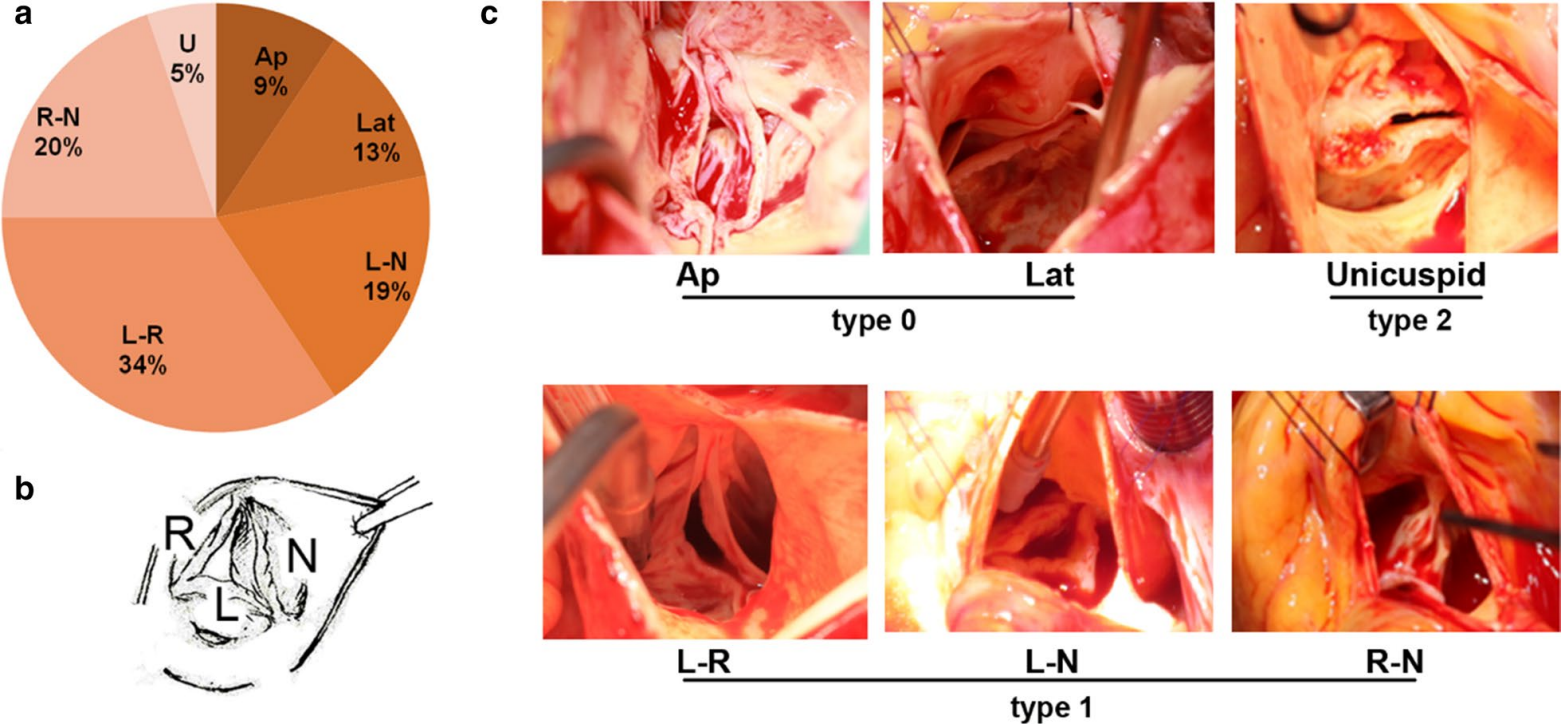
The bicuspid aortic valve (BAV) classification. **a** composing rate of BAV in this study, **b** the aortic valve anatomic sketch (photographer’s view), **c** the BAV types are confirmed during surgeries. *Ap* anterior–posterior, *Lat* lateral, *L* left coronary sinus, *N* non-coronary sinus, *R* right coronary sinus, *U* unicuspid

From January 2015 to December 2019, 130 unrelated patients with BAV were continuously recruited from the division of cardiothoracic and vascular surgery of Tongji hospital. Patient’s phenotypes of BAV were detected by echocardiographic examination, and confirmed by the cardiac surgeon during the operation. The pattern of BAV, as displayed in Fig. 1, was recorded according to the phenotype category described by Sievers et al. [11]. The inclusion criteria were patients with BAV underwent cardiac surgery in our institution and agreed to participate in the trial. The exclusion criteria consisted of unidentifiable valve phenotype, complex congenital heart disease, and malignant tumors. Finally, a total of 96 surgical patients were included in this study. Informed consents were acquired from all the participants. The local research ethics committee approved the study.

**Fig. 2:**
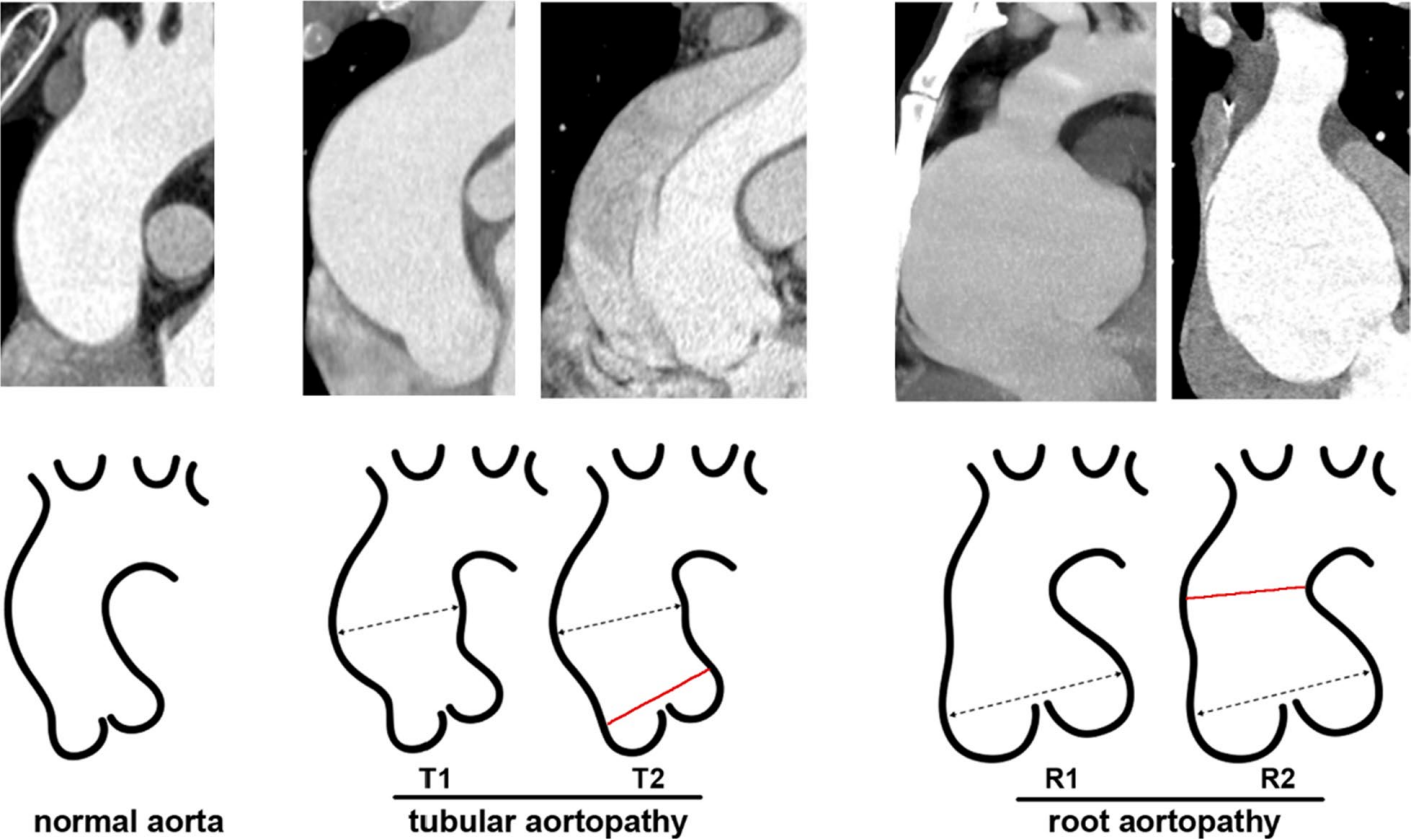
Aortopathy classification. The dotted line marks the widest part of the aorta. The red line indicates the dilated segment. T1, ascending aorta dilatation with normal aortic root; T2, ascending aorta dilatation with aortic root expansion; R1, aortic root dilatation with normal ascending aorta; R2, aortic root dilatation with ascending aorta expansion

The aortic diameter was measured at least at 2 levels: sinus of Valsalva and the widest level of the ascending aorta. According to the 2014 European Society of Cardiology guidelines, the aortic diameter stretching 40 mm or greater is considered as aortopathy [12]. For patient with suspected aortopathy, computed tomography angiography was further required to evaluate the geometrical morphology of the ascending aorta. The root type aortopathy was defined as the largest diameter located in the aortic sinus. The tubular type aortopathy was defined as the largest segment located in the ascending aorta. The aortopathy classification was illustrated in Fig. 2.

### Targeted NGS

A custom panel including 13 BAV genes was designed using Ion AmpliSeq™ designer software. The gene selecting criteria were causal genes of BAV and genes highly correlated to BAV aortopathy. The gene list and PubMed unique identifier of references were listed in Table 1. Genomic DNA was extracted from peripheral blood leucocytes using a blood DNA kit (Tiangen Biotech, Beijing, China). 96 samples were genetically evaluated using targeted resequencing on the Ion Proton platform (Life Technologies).

**Table 1:** Genes in the panel and rare variants burden comparison between patients and ExAC controls

Gene	PMID	Number of variants in 192 patients' alleles	Allele count in ExAC East Asia group	*P* value
*GATA6*	29653232	3	19 in 7684	0.016
*GATA5*	24796370	2	7 in 7858	0.018
*NOTCH1*	16025100	9	130 in 7778	0.006
*TGFBR1*	25145529	1	11 in 8132	0.244
*TGFBR2*	23578328	2	31 in 8228	0.173
*FBN1*	24564502	5	132 in 8614	0.227
*ELN*	14666267	2	68 in 8224	0.675
*EGFR*	26708639	1	61 in 8592	> 0.999
*FLNA*	21815255	2	122 in 6610	> 0.999
*ACTA2*	17994018	0	10 in 8432	N.A
*GATA4*	21330551	0	144 in 7658	N.A
*NKX2-5*	25438918	0	10 in 7678	N.A
*SMAD6*	22275001	0	29 in 8050	N.A

*ExAC* The Exome Aggregation Consortium, *PMID* PubMed unique identifier of reference, *N.A.* not available

### Data analysis and filtering

Sequencing data were processed with Ion Torrent Suite Software v5.0 (Life Technologies) to align reads to the human genome reference (hg19/GRCh37). The filtered variants with allele frequency < 0.05% in the online databases 1000 Genomes Project or the Exome aggregation consortium (ExAC) [13, 14] were selected as rare variants. We applied computational prediction algorithms: SIFT, Provean, PolyPhen-2 [15], and CADD [16] to predict the functional significance of the identified rare non-synonymous coding substitutions; Phylop scores to measure evolutionary conservation. Finally, we defined pathogenicity of variants in accordance with the American College of Medical Genetics and Genomics (ACMG) guidelines [16].

The rare variants fund by NGS were then validated on an automated DNA sequencer ABI 3130 XL (Applied Biosystems, Foster City, CA) using the Big Dye Terminator v3.1 kit (Thermo Fisher Scientific, Waltham, MA, USA) following the manufacturer’s guidelines.

### Copy number variants (CNVs)

To detect potential large genomic structural variation such as deletions, duplications and other CNVs, the copy number of all sequenced regions was analyzed by amplicon coverage data.

### Rare variant burden analysis

To assess the contribution of genetic variant risk to BAV, the frequency of qualifying rare variants per gene in our patient’s cohort were then compared to that in the ExAC East Asian database. The controlling variant was all nonsynonymous and loss-of-function variants with allele frequency < 0.05% among ExAC data base. The results of rare variant burden were list in Table 1.

### Statistical analysis

Continuous variables were presented as mean ± standard deviation, and compared using the t-test or the Mann–Whitney U-test. The one-way ANOVA test was employed for multigroup analysis. The post-hoc tests were processed in the pairwise comparisons manner. Categorical variables were expressed as percentages, and analyzed using the Fisher’s exact test or the Chi-square test. The post-hoc test of 2 × 3 contingency table was performed with adjusted standardized residual analysis. The mosaic plot was employed to illustrated the relationship between aortic configuration and rare variants. The cell represented positive relationship between two variables was for blue shades. The multivariable logistic regress model was built for aortopathy risk factor analysis. All two-tailed *P* < 0.05 was considered statistically significant. All statistical analyses were performed with R version 3.2.5 (Vienna, Austria).

## Results

### The demographic characteristics

The study enrolled 96 individuals. Of them, 74 males and 22 females, with a mean age of 44.6 ± 13.0 years (ranged from 15 to 67 years). The aortic dilatations were found in 34 patients (35.4% of all the participants), of them, 22 patients underwent surgery for the aortic aneurysm or dissection. In this cohort, no aortic coarctation was observed. With regard to BAV type, left–right model is the most common type found in one-third of the BAV patients (34.4%).

### Sequencing data

The NGS was offered to all of the 96 participants. The average depth of coverage for the targeted regions was 1406 reads. The percentage of sequencing on target was 93.77%. Greater than 50 × coverage was obtained for 99.01% of the bases sequenced. A total of 27 rare nonsynonymous coding variants involving 9 genes were identified by targeted capture in 25 BAV patients. Two patients harbored 2 rare variants, respectively. The variants comprised 24 missense, 1 frameshift, and 2 nonsense mutations. Of them, 14 rare variants had been reported in neither ExAC nor ClinVar database previously. The details of rare variants were presented in Additional file 1: Table S1.

The potential CNVs were validated by real-time polymerase chain reaction but no large deletion or duplication was detected.

### Rare variant burden analysis

Of the 13 BAV genes, the frequency of rare nonsynonymous coding variants of genes *GATA5*, *GATA6*, and *NOTCH1* were significant higher in the 96 BAV cohort compared to the ExAC online database.

### Genotype–phenotype correlation

The patients were compared between rare variants group and no variants group. There was no significant difference of variables between the two groups as depicted in Table 2.

**Table 2:** Demographic and clinical characteristics of the bicuspid aortic valve

Variables	No variant (n = 71)	Variant (n = 25)	*P* value
Age (years)	45.6 ± 12.7	41.5 ± 13.7	0.173
Male (%)	58 (81.7)	16 (64.0)	0.070
BMI (kg/m^2^)	22.0 ± 3.4	23.1 ± 4.8	0.242
Hypertension (%)	23 (32.4)	10 (40.0)	0.491
Diabetes mellites (%)	9 (12.7)	4 (16.0)	0.737
Bicuspid valve type (%)		0.524	
Anterior–posterior	8 (11.3)	1(4.0)	–
Lateral	8 (11.3)	4 (16.0)	–
L-N	13 (18.3)	5 (20.0)	–
L-R	22 (31.0)	11 (44.0)	–
R-N	15 (21.1)	4 (16.0)	–
Unicuspid	5 (7.0)	0	–
Aortic valve disease (%)		0.983	
Aortic insufficiency	27 (38.0)	10 (40.0)	–
Aortic stenosis	18 (25.4)	6 (24.0)	–
Combined lesions	26 (36.6)	9 (36.0)	–
Infective endocarditis (%)	13 (18.3)	4 (16.0)	> 0.999
Aortopathy (%)	23 (32.4)	11(44.0)	0.297
Aortic dissection (%)	4 (5.6)	3 (12.0)	0.372
Surgery for aortopathy (%)	15 (21.1)	7 (28.0)	0.482
LVEF%	60.0 ± 9.2	58.6 ± 10.8	0.521

*BMI* body mass index, *LVEF* left ventricular ejection fraction, *L* left coronary sinus, *N* non-coronary sinus, *R* right coronary sinus, *SD* standard deviation

**Fig. 3:**
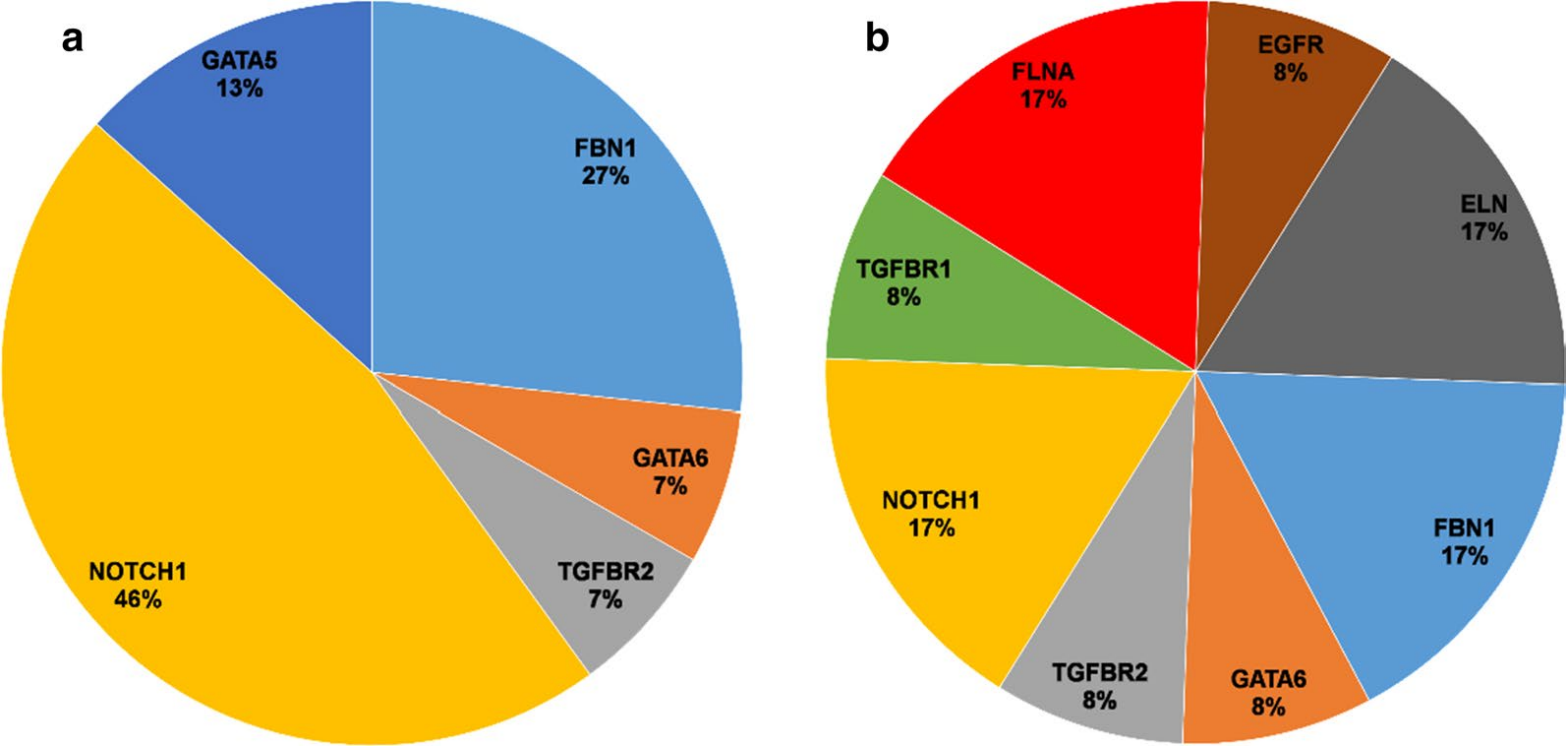
The spectrum of rare variants detected in patients of normal aorta (**a**), and aortopathy (**b**)

We then investigated the distribution of rare variants in the normal aorta group and aortopathy group (including both the root and tubular types). The variants number were comparable in two groups, i.e. 15 in normal group and 12 in aortopathy group, respectively. Interestingly, the constituent ratios of rare variants displayed distinct distribution characteristics between the two groups. In the normal aorta group, the frequency of *NOTCH1* variant reached 46.7% of all detected rare variants. Oppositely, the variants in *NOTCH1* accounts for 16.7% of all detected variants in aortopathy group, though the difference was not statistically significant (*P* = 0.217). Meanwhile, the number of involved genes in aortopathy group was more than that in the normal group (8: 5 genes). The rare variants distribution was illustrated in Fig. 3. This suggested that the BAV aortopathy may have a more complex genetic background than the normal group.

### BAV aortopathy

One third of the BAV patients (35.4%, 34/96) were diagnosed with aortopathy. We compared patients among normal aorta, tubular aortopathy, and root aortopathy groups. The patients of tubular aortopathy were the oldest, with a mean age of 53.6 years. In contrast, the aortic root aortopathy group was the youngest. The number of tubular type cases was 4 times as high as root type (27:7). The aortic dissection was more often in root group (28.6%, 2/7) than in normal (3.2%, 2/62) and tubular (11.1%, 3/27) groups (*P* = 0.048). Of note, none in root group had aortic calcification or infective endocarditis. The comparative results were presented in Table 3.

**Table 3:** Comparing aortopathy and normal ascending aorta

Variables	Normal aorta (n = 62)	Tubular aortopathy (n = 27)	Root aortopathy (n = 7)	*P* value
				Total
Onset age (years)	41.7 ± 12.7	53.6 ± 8.9 *	35.0 ± 11.8	< 0.001
Male (%)	40 (75.8)	22 (81.5)	5 (71.4)	0.878
Diabetes mellitus (%)	6 (9.7)	5 (18.5)	2 (28.6)	0.102
Hypertension (%)	24 (38.7)	9 (33.3)	0	0.081
Variant burden (%)	14 (22.6)	6 (22.2)	5 (71.4) ^†^	0.018
Aortic dissection (%)	2 (3.2)	3 (11.1)	2 (28.6)	0.024
Infective endocarditis (%)	14 (22.6)	3 (11.1)	0	0.070
Multiple valves replacement (%)	15 (24.2)	1 (3.7)	1 (14.3)	0.070

^*^With the ANOVA test for the onset age, statistically significant difference was found between normal aorta, tubular aortopathy and root aortopathy groups. Tubular group is the oldest according to the post-hoc test

^†^For variant burden, Chi-square test indicated a statistically significant difference between three groups. Adjusted residual for root group was 2.3 (*p* = 0.00045), reaching the threefold Bonferroni-adjusted significance level of *p* < 0.0167)

**Fig. 4:**
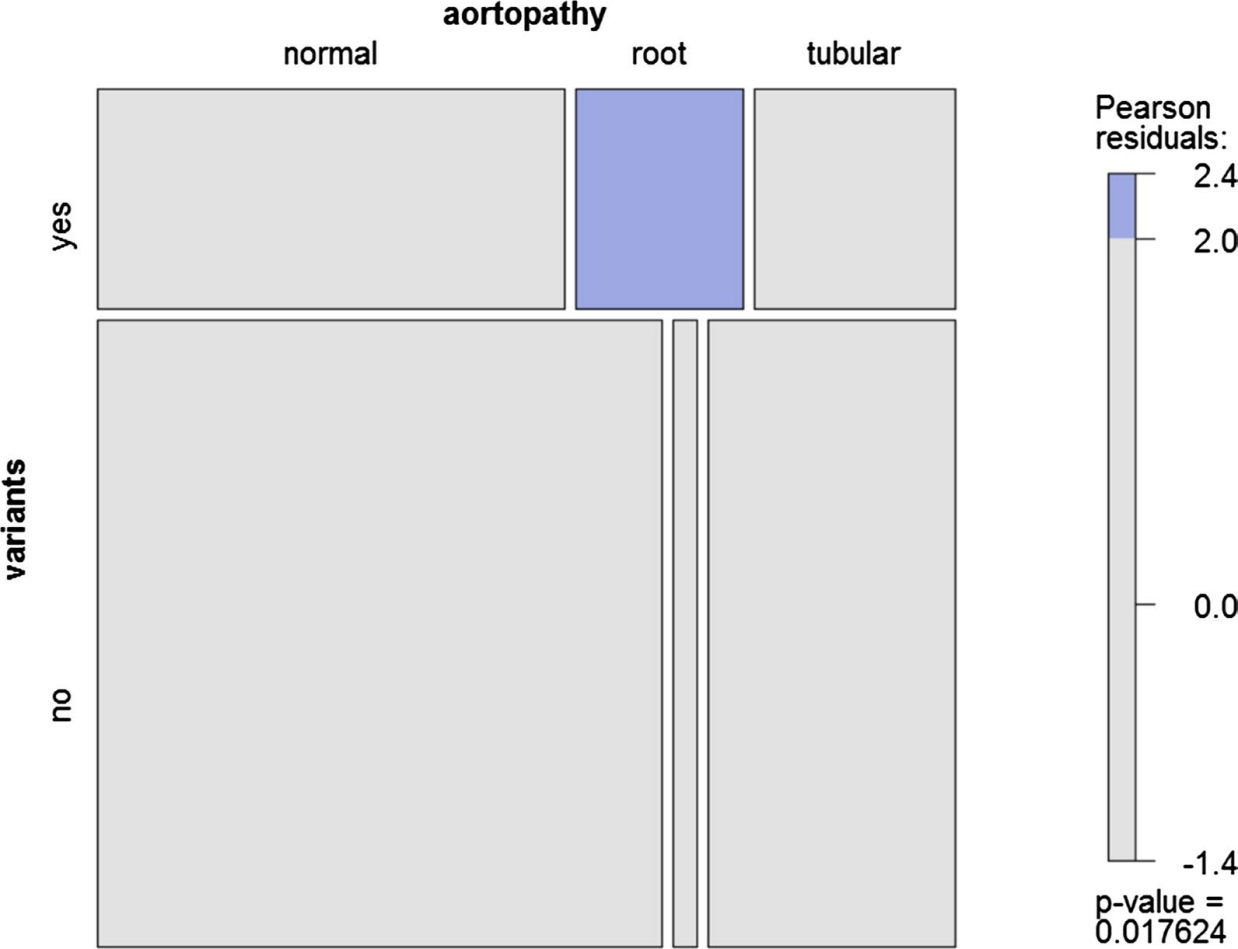
Mosaic plot represents the positive relationship between rare variant and root aortopathy

With regard to the genetic influence of the BAV aortopathy, there were 5 (71.4%) patients in the root group harbored rare variants. Instead, number of rare variant case was 8 (29.6%) in tubular group and 18 (29.0%) in normal group, respectively. The patients of root type aortopathy had the highest frequency of rare variants among the BAV patients (*P* = 0.021). In the mosaic plot (Fig. 4), the cell of rare variant and root aortopathy was for blue with the Pearson's residuals of 2.8, represented a positive relationship between the rare variant number and root aortopathy cases. In addition, a logistic-regress model was built for risk factors of root aortopathy, by including variables of rare variant, gender, age, body surface area, hypertension, aortic valve disease (insufficiency, stenosis, and combined lesions) and BAV type (type 0,1,2). The rare variant was identified as the independent risk factor [*P* = 0.014, hazard ratio = 23.9, 95% confidence interval (1.9–302.9)].

## Discussion

In the present study, we reported on targeted resequencing detection of the rare variants associated with BAV and related aortopathy. With a 13-gene panel, an overall rare variants detection rate of 26.0% in 96 patients was achieved. To the best of our knowledge, this study was the largest cohort by targeted resequencing accessing the pathogenic variants associated with sporadic BAV disease in the Eastern Asian population. According to our data, the BAV patients with aortopathy displayed more complex genetic heterogeneity than BAV patients with normal aorta. The root type aortopathy was strongly associated with the rare variants of BAV genes.

The flourishing availability of NGS technologies provided us the convenience to rapidly detect multiple variants in multiple genes, by which, a more comprehensive understanding of the heritability of the BAV and associated aortopathy would be reached. Bonachea et al. conducted a targeted sequencing assay of 78 unrelated BAV patients [17]. They identified 31 putative disease-causing variants in 16 individuals. Dargis et al. used a panel of 9 genes associated with BAV to study 48 patients [18], they identified 19 potentially pathogenic variants. Of them, 7 variants in *NOTCH1* took the highest burden of deleterious variants. Some researchers also reported the NGS results of the BAV aortopathy. Girdauskas et al. used a 20 genes panel to investigate 63 BAV patients with root aortopathy [19]. A total of 64 rare variants in 15 candidate genes were identified, of them 24 were potentially pathogenic/likely pathogenic variants. Gillis used a panel of 22 genes to study a large BAV/ thoracic aortic aneurysm cohort [10]. They identified 112 variants classified as pathogenic in 441 patients. In comparison with these previous studies, our cohort reaches a similar rare variants detection rate, suggests that the BAV might be a combination as a polygenic trait, many discrete genes being responsible for the disease.

Some studies have suggested that aortic dilatation of BAV is an inherent attribute of the disease. In a long-term follow-up research, the accelerated aortic dilatation in BAV was observed even independent of severe aortic stenosis or regurgitation and aortic coarctation [4]. Although the mechanism of aortic dilation has been long debated, the genetic theory was continuously advocated and investigated. Based on genome-wide single nucleotide polymorphism array, the early onset of thoracic aortic disease was demonstrated frequently in BAV patients, and associated with recurrent rare copy number variations [20]. These findings suggest that BAV and aortopathy might share some common molecular mechanism, the genetic defect may hurt the cardiac or vascular development, leading to the early onset of disease.

Several recent studies focused on the genic origin of BAV related aortopathy. Pepe et al. suggested *FBN1* was an important cause of the aortic root dilatation in BAV [21]. Gillis et al. [10] considered variants in *SMAD6* has a significant contribution to BAV aortopathy, with a variant burden of 2.5%. In our study, there is a wide spectrum of rare genetic variants in patients with aortic dilatation, no gene plays an outstanding role.

*NOTCH1* is the first gene proved to be associated with both familial and sporadic BAV cases. Altered Notch signaling causes malformations of the left ventricular outflow tract including BAV and facilitates the progress of aortic valve disease [22]. Controversy also exists in whether the *NOTCH1* variant is a causal of BAV aortopathy. Girdauskas et al. found a wide spectrum of the variants in 19 of 63 patients with BAV root aortopathy, and *NOTCH1* was the leading causal gene [19]. In contrast, Kent et. al investigated BAV related aortopathy by sequencing the *NOTCH1* gene in 13 BAV families. They failed to identified causal variant in the patients and concluded that the *NOTCH1* variant contributed little to the noncalcified bicuspid aortic valve combined with ascending aortic aneurysm [23]. Ambitiously, in another multiple center sequencing study, *NOTCH1* was suggested as a protective factor for BAV related aortic aneurysm [10]. In our cohort, 9 nonsynonymous rare variants in *NOTCH1* were identified in unrelated patients, with the detection rate of 9.4%. The phenotypes of rare variant carriers were distinct from each other. Generally, the *NOTCH1* variants were more common in the phenotype of valve dysfunction with normal aortic shape than the phenotype of aortopathy. Our findings support the argument that *NOTCH1*-dependent mechanism has a heterogeneity and low penetrance of aortic aneurysm in the BAV patient. Still, *NOTCH1* plays the important role in the pathogenesis of BAV and in its complications.

According to the previous study and our data, the incidence of root aortopathy is ranged from 7.3 to 13.5% in patients with BAV [24]. This type of aortopathy has a tendency of rapid progress and high risk of adverse aortic events [24, 25]. Since the root aortopathy presents a marfanoid-like aortic root morphology, it is reasonable to suggest that the genetic factors may determine a defect of the aortic wall, leading to the aortic root dilation. In our study, the pathogenic variants in *TGFBR1*, *TGFBR2*, *FLNA*, and *FBN1* are responsible for the aortic root dilatation. Indeed, it is a compelling finding that the pathogenic variants accounted for 57.1% of root phenotype in this consecutive BAV cohort.

The *FBN1* variant has been associated with syndromic or no syndromic bicuspid aortic aneurysm [21, 26]. Meanwhile, the *FLNA* defects have been demonstrated to cause various developmental malformations involving the brain, skin, bone, and cardiovascular. According to Chen et al. [27], the BAV is one of the most common cardiac malformations occurred in 5.3% of the filaminopathy patients. In our group, the majority of root group patients are lack of typical systemically connective tissue disease presentations. The explanation may be the reduced penetrance of the genetic variants.

Since the phenotypes are overlapped and with low penetrance of some variants, it is challenging to make differential diagnoses with BAV related aortopathy such as fibrillinopathy and filaminopathy by only clinical manifestation. The insight of BAV genetic background helps to improve the understanding of the pathology of these diseases. In view of the fact that rare variants in some special genes act as potential modulator of BAV and associated aortopathy, the molecular diagnosis can refine the accuracy of clinical diagnosis, and thereby provides optimal guidance on patient’s management and helps to develop more tailored therapeutic strategies.

## Limitation

Firstly, our participants were all recruited from the surgical department. There would be a potential bias of assessing genetic effect to represent the general population. Secondly, our study method focused on the impacts of the rare variants of known BAV genes on the disease. Whole-genome sequencing in a larger cohort might be helpful to broaden genetic horizon of BAV and relative complications. The variants would also need further functional studies to evaluate the relevance. Long-term follow-up is also warranted for assessing the potential pathogenicity of the rare genetic variants.

## Conclusions

In conclusion, this targeted NGS approach revealed a wide and specific gene variant spectrum in a large cohort of patients with BAV. From the insight of the genetic view, it is reasonable to classified the aortopathy as tubular and root type. The root phenotype presented high pathogenic variant burden among BAV patients. Patients with rare nonsynonymous coding variants had an accelerated disease progression and underwent surgical management at an early age. The molecular evaluation of BAV disease could help to accurate the diagnosis and was worth further researched.

## Supplementary Information

Additional file 1: Table S1. Rare variants identified in BAV patients.

## Data Availability

The dataset supporting the conclusions of this article is available in the NCBI Sequence Read Archive (SRA) repository, https://dataview.ncbi.nlm.nih.gov/object/PRJNA724868?reviewer=f85ctpn3hrjhi1jgmbr3dthip8

## Abbreviations

ACMG: The American College of Medical Genetics and Genomics
BAV: Bicuspid aortic valve
CNVs: Copy number variants
ExAC: The Exome aggregation consortium
NGS: Next generation sequencing

## Declarations

### Statement of methods

All methods in this study were carried out in accordance with relevant guidelines and regulations.
