# Supplementary material for: Aortic root aortopathy in bicuspid aortic valve associated with high genetic risk
Source: BMC Cardiovasc Disord. 2021 Aug 30;21:413. doi: 10.1186/s12872-021-02215-y (PMC8404252; doi:10.1186/s12872-021-02215-y)
Supplement: Supplementary file 1 — Rare variants identified in BAV patients. [file 12872_2021_2215_MOESM1_ESM.pdf]

Supplemental table 1. rare variants identified in BAV patients

Gene	Position	Type	Function	dbSNP ID	Protein	Coding
EGFR	chr7:55273254	SNV	missense	N.A.	p.Glu1193Lys	c.3577G>A
ELN	chr7:73470642	SNV	missense	rs2229427	p.Gly398Arg	c.1192G>A
ELN	chr7:73467631	SNV	missense	rs571843793	p.Ala363Val	c.1088C>T
FBN1	chr15:48704767	SNV	missense	novel	p.Glu2742Gly	c.8225A>G
FBN1	chr15:48712949	SNV	missense	novel	p.Ile2585Thr	c.7754T>C
FBN1†	chr15:48720648	SNV	missense	novel	p.Lys2298Glu	c.6892A>G
FBN1	chr15:48741042	SNV	missense	novel*	p.Cys1865Tyr	c.5594G>A
FBN1‡	chr15:48725143	SNV	missense	novel	p.Arg2220Gln	c.6659G>A
FLNA	chrX:153587625	INS	ins	novel*	p.His1431fs	c.4291_4292insC
FLNA	chrX:153594950	SNV	missense	rs370490152	p.Glu349Lys	c.1045G>A
GATA5	chr20:61041544	SNV	missense	novel	p.Arg255Trp	c.763C>T
GATA5‡	chr20:61050250	SNV	missense	novel	p.Gly110Cys	c.328G>T
GATA6	chr18:19751656	SNV	missense	rs387906816	p.Ser184Asn	c.551G>A
GATA6	chr18:19752095	SNV	missense	novel	p.His330Gln	c.990C>A
GATA6	chr18:19751308	SNV	missense	rs200483324	p.Glu68Val	c.203A>T
NOTCH1	chr9:139396455	SNV	missense	novel	p.Arg1824Trp	c.5470C>T
NOTCH1	chr9:139401379	SNV	missense	novel	p.Asp1230Glu	c.3690C>A
NOTCH1	chr9:139412303	SNV	nonsense	novel*	p.Arg448Ter	c.1342C>T
NOTCH1	chr9:139409131	SNV	missense	N.A.	p.Asp680Asn	c.2038G>A
NOTCH1	chr9:139399315	SNV	missense	rs369467132	p.Ala1610Ser	c.4828G>T
NOTCH1	chr9:139401375	SNV	missense	N.A.	p.Val1232Met	c.3694G>A
NOTCH1	chr9:139401321	SNV	missense	novel	p.Gly1250Ser	c.3748G>A
NOTCH1	chr9:139402516	SNV	missense	rs374230681	p.Gln1134Arg	c.3401A>G
NOTCH1	chr9:139411768	SNV	missense	rs201768800	p.Arg504His	c.1511G>A
TGFBR2†	chr3:30691797	SNV	missense	novel	p.Val125Ala	c.374T>C
TGFBR2	chr3:30729962	SNV	missense	rs104893819*	p.Arg520Ter	c.1558C>T
TGFBR1	chr9:101911535	SNV	nonsense	rs113605875*	p.Arg487Gln	c.1460G>A

*indicates pathogenic variant; ‡ and † indicate the variants in the same carrier corresponding to the symbol, respectively. dbSNP, the Single Nucleotide Polymorphism database; fs: frameshift; INS/ins, insertion; SNV: single nucleotide variant
